# High protein intake without concerns?

**DOI:** 10.1186/s13054-017-1699-9

**Published:** 2017-05-15

**Authors:** Olav Rooyackers, Martin Sundström Rehal, Felix Liebau, Åke Norberg, Jan Wernerman

**Affiliations:** 1 Department of Anesthesia and Intensive Care Medicine, Karolinska University Hospital Huddinge, Stockholm, Sweden; 2 Division of Anesthesia and Intensive Care Medicine, CLINTEC, Karolinska Institutet, Stockholm, Sweden

**Keywords:** Protein intake, Protein turnover, Nitrogen balance, Protein balance, Critical care

## Abstract

The high fashion in nutrition for the critically ill is to recommend a high protein intake. Several opinion leaders are surfing on this wave, expanding the suggested protein allowance upwards. At the same time, there is no new evidence supporting this change in recommendations. Observational data show that in clinical practice protein intake is most often far below current ESPEN recommendations of 1.2–1.5 g/kg/day. Therefore, it may be in the best interests of our patients just to adhere to that guideline, and not to stretch them upwards for protein intake? Here we give arguments to stay conservative.

## Background

Evidence behind recommendations for nutrition in the critically ill is problematic. This relates mainly to four areas: 1) the limited physiological knowledge of the regulation of protein turnover, in particular in the critically ill; 2) the paucity of prospective randomized controlled trials (RCTs) on protein delivery; 3) the heterogeneity of the patient group referred to as critically ill; and 4) the time course of critical illness. Recommendations given may be feasible and beneficial for some patients, while the same recommendation may inflict dangers or even harm to others. Therefore “individualized nutrition” is a concept launched by several authors to overcome some difficulties, but an individualized guideline immediately creates new problems. The major difficulty is the absence of a clinically available and practical technique to monitor the efficacy and potential harm of protein intake. From a personal perspective, we here discuss protein intake in the critically ill, its evidence, rationale, physiology, and potential dangers.

There seems to be rather solid observational evidence supporting the statement that a larger muscle mass at intensive care unit (ICU) admittance is associated with a more favorable outcome [1]. This may also be one explanation for the so-called ‘obesity paradox’ in critically ill patients, as overweight people often also have a large muscle mass. Furthermore, the development of muscle protein depletion is associated with mortality outcomes as well as quality of life-associated outcomes following critical illness [2, 3]. Therefore, efforts to attenuate the depletion of muscle mass and body protein mass may be beneficial. For muscle protein the rate of depletion is most pronounced in the early phase of critical illness [4, 5]. The rate of depletion then subsides for the patients staying in the ICU for a prolonged period of time (Gamrin et al., unpublished data).

All existing guidelines for protein intake in the critically ill give reference to the fairly large number of published observational studies on protein intake in ICU patients. Most often a larger protein intake is associated with a more favorable outcome. However, whether this is due to the fact that it is clinically easier to feed less ill patients or whether it actually prevents muscle loss is an unanswered question. There is one old observation that a shift from amino acid net export to uptake of provided amino acids into muscle tissue after a week of critical illness is a favorable prognostic sign [6], but is this attributable to nutrition or to recovery? Furthermore, as many commercial products contain a fixed proportion of calories to protein, a difference between energy intake and protein intake in relation to outcomes becomes visible only when energy intake is adjusted to energy expenditure rather than some measure of body size [7]. Overall, for both energy and protein intake, in general clinical practice there is under-nutrition, even in relation to very conservative guidelines [8, 9].

## Physiology

Protein metabolism differs between tissues and also between individual proteins in the individual cell; in addition there may be a time-related pattern with a circadian rhythm. On the other hand, protein intake is for the whole body, usually delivered as meals by the gastrointestinal tract.

During critical illness there is an increase and a shift of protein synthesis towards processes that are life-saving in the immune system, in the liver, and at the site of injury, etc. [10, 11]. Simultaneously, there is an increase in protein degradation, also with an altered focus, primarily in skeletal muscle [12]. Existing quantitative data usually reflect mixed protein turnovers, which may give an incomplete picture of the processes. Existing techniques to monitor protein turnover are not easily applicable to critically ill subjects in clinical practice outside specific study protocols. Overall, this limited knowledge of the regulation of quantities of individual protein as well as mixed protein in tissues or in the whole body make it difficult to decide on what grounds recommendations of protein intake should rest. At a whole body level, and as a group, critically ill patients utilize extra nutritional proteins to build body protein and they do not oxidize these [11, 13–15], but due to the black box principle it is not clear which protein are synthesized and whether they benefit the patient.

## Paucity of RCTs

There are observational studies of case series character, meaning that there is no protocol to which patients are randomized. Rather, there is a post-hoc organization of subjects into cohorts. Studies of total body protein using neutron activation come into this category and demonstrate that a protein intake of 1.47 g/kg/24 h is better than 1.14 g/kg/24 h, but that 1.86 g/kg/24 h adds no additional advantage [16]. Also, in terms of mortality outcomes, 1.46 g/kg/24 h is reported as advantageous compared to 1.06 g/kg/24 h and 0.79 g/kg/24 h [7]. Furthermore, short-term nitrogen balance studies report an improved nitrogen economy, where a protein intake of 1.7–2.2 g/kg/24 h is superior to lower intakes, and an intake of 2.7 g/kg/24 h may even attain a nitrogen economy in balance in critically ill subjects [17].

Another category of observational studies are register extracts, again with post-hoc categorization. These reports have revealed that in general clinical practice there is an often severe under-nutrition even in relation to very conservative guidelines for both energy and protein intake [8, 9, 18–21]. An improved mortality outcome related to both a higher calorie and protein intake is reported for both low and high body mass index (BMI) cohorts [8]. A protein intake of >1.2 g/kg/24 h is reported to be associated with a better mortality outcome as compared to an intake of <1.0 g/kg/24 h [19]. However, this observation was confined to non-septic patients. In the same study, an energy deficit of 10–20% related to measured energy expenditure was associated with the best mortality outcome. A similar separation between calorie and protein intake on mortality outcome was recently reported in a multicenter observational study, where an intake >80% of prescribed protein (which can be deduced to be around 1.0 g/kg/24 h), but not a similar success rate of energy intake, were associated with a better mortality outcome [20]. In a second publication, the investigators report that this advantage is most obvious in subjects staying for >12 days and with a high nutrition risk score [21].

In a high-quality observational study, all patients that fulfill inclusion criteria should be included, and they should be appropriately characterized. Internal and external validity of observations should be specified. Finally, the inherent limitation that an observational study, by definition, can only produce hypothesis-generating conclusions should be emphasized. Sometimes observational studies result in guideline recommendations, which is to stretch conclusions beyond scientific standards.

In existing prospective randomized studies the generalizability is not obvious. The classic nitrogen balance study shows a cumulated improvement in whole body nitrogen economy for a protein intake of 1.2 g/kg/24 h as compared to a lower intake, but no further improvement in nitrogen economy above that level of intake [22]. A recent randomized comparison between 0.8 and 1.2 g/kg/24 h over 7 days reports improved handgrip strength and thigh muscle thickness, with a marginal difference in nitrogen balance [23]. In another study, for the purpose of preventing kidney injury, critically ill patients were randomized to receive extra intravenous amino acids, giving a comparison between 0.75 and 1.75 g/kg/24 h of protein intake [24]. The study revealed no differences between the two groups in terms of duration of renal failure or any other outcome parameters related to critical illness.

## The heterogeneity

Despite similarities in organ failure, treatment modalities, and the high staff density in ICUs, there are considerable differences in diagnoses and prognosis of critically ill patients. Regarding guidelines and interpretation of study results, these differences become quite obvious and problematic. Regarding optimal protein feeding and the available evidence, we find that inclusion and exclusion criteria reflect this heterogeneity and make comparisons of different studies difficult.

Inclusions may be confined to subjects on parenteral nutrition only [13, 23, 25, 26], BMI >17 [27], no signs of liver failure [17, 28], mechanical ventilation [13, 17, 19, 28], enteral nutrition only [20, 21, 29], no diabetes [17, 28, 29], and access to indirect calorimetry data [7, 13, 17, 28], etc. Although most journals demand a CONSORT diagram over screened and included patients, many studies do not communicate the level of selection that has preceded the screening. This is particularly true for register studies, where access to complete datasets or availability of particular data becomes one of the most important inclusion criteria.

It is well known that mortality outcomes vary with gender, even with similar risk scoring. The comparability between groups, whether randomized or somehow dichotomized, is most often documented by identity of anthropometric data and risk scoring, or organ failure scoring. This is usually what is possible, but it should be realized that the predictive value of risk scoring or organ failure scoring is validated in large unselected cohorts of critically ill patients, which may correspond poorly to the subjects in a particular study. It has been pointed out that, from a nutrition perspective, critically ill patients in the ICU may be divided into three categories: 1) those who are likely to recover regardless of nutrition treatment; 2) those who are likely to have an unfavorable outcome regardless of nutrition treatment; and finally 3) the group for whom nutrition treatment may make a difference [30]. The three groups may be difficult to separate in clinical practice, but if groups 1 and 2 constitute a major proportion in a study population, a possible treatment effect is highly likely not to be detected.

## The time course

Another confounder when interpreting existing evidence is the time perspective of nutrition in the critically ill. Most studies include patients during the initial period of ICU stay, which represents the acute phase of critical illness. Post-hoc analyses of the EPANIC study report disadvantages in terms of time in ICU related to both energy and protein intake [27, 31]. In observational studies, favorable effects on mortality outcomes are confined to the period post-day 4 or post-day 12 of ICU stay [19–21]. Despite the observation that a cumulated energy deficit or protein deficit is most often attributable to a low intake during the initial phase of critical illness [32, 33], the reports of positive effects of a high or enhanced intake during this period are sparse.

## Relation to protein turnover

In a pediatric study of infants undergoing cardiac surgery, patients were randomized to receive 2 g/kg/24 h or 5 g/kg/24 h, and the effects on protein kinetics were evaluated [26]. The investigators report no differences in the protein kinetics studied, but reported a higher amino acid oxidation and a higher blood urea nitrogen in the high-protein group. They emphasize the risk for a metabolic acidosis in relation to the oxidation and ureagenesis. In another pediatric study, septic adolescent subjects received a protein intake of 1.5 g/kg/24 h or 3.0 g/kg/24 h in a cross-over protocol [25]. The investigators report an improved protein balance mainly attributable to an effect on whole body protein synthesis; at the same time, however, there was also an elevated rate of amino acid oxidation.

**Fig. 1:**
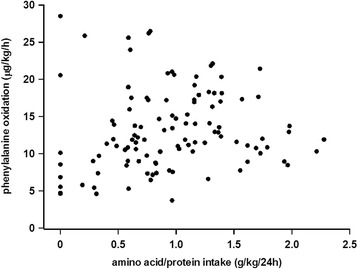
The measured oxidation of phenylalanine during measurements of whole body protein turnovers in critically ill patients. The energy and protein intakes were constant in the individual subjects but not protocolized. All measurements were performed after short-term (<48 h) nutrition exposures. From published data in [11, 13–15]

**Fig. 2:**
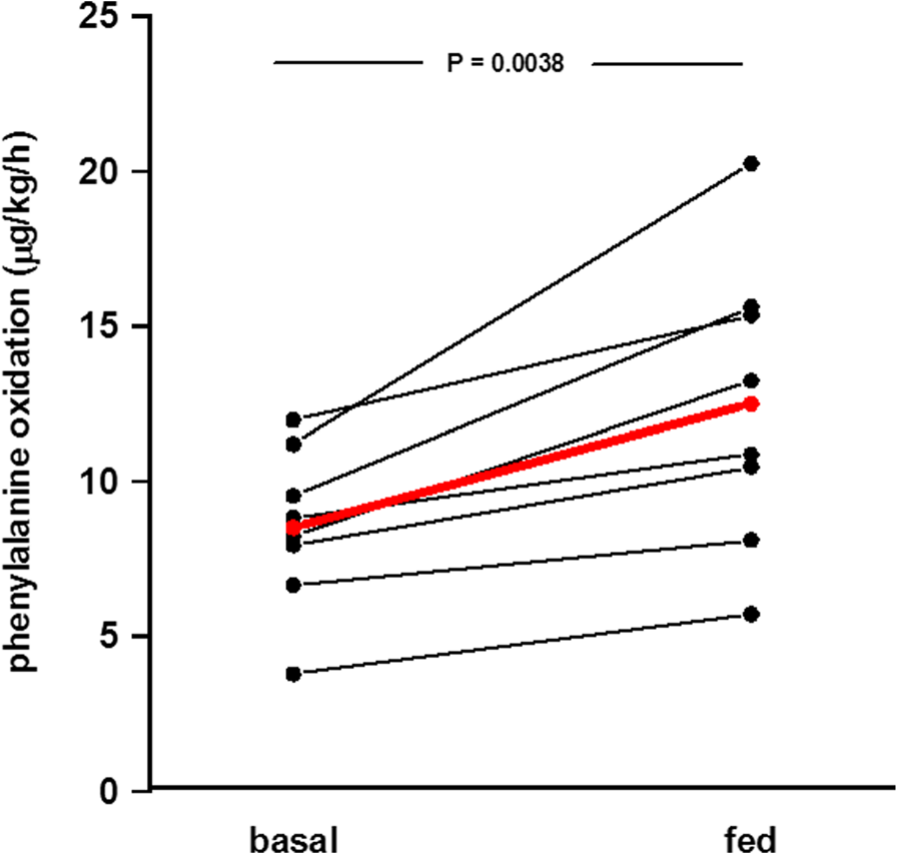
The measured oxidation of phenylalanine during measurements of whole body protein turnovers in healthy volunteers. At baseline there was zero intake in the post-absorptive state. In the fed state, the subjects had been given parenteral nutrition corresponding to their energy expenditure including protein corresponding to 1.0 g /kg/24 h for 4 h. From published data in [11]

In studies on whole body protein turnover in adult critically ill patients from our own research group, no increase in amino acid oxidation is reported during short-term studies (<48 h) (Fig. 1), with a protein intake up to 2.0 g/kg/24 h [11, 13, 14]. In parallel, a dose-related improvement in whole body protein balance is seen. These studies were performed during the initial phase of critical illness (first week of ICU stay), with a cross-over protocol but without a nutrition protocol beyond control of protein intake. The questions of an increase in amino acid oxidation and a hypothetic associated risk of metabolic acidosis when increasing the protein intake, although controversial, need more attention [26, 34]. In contrast to the critically ill patients, healthy volunteers show an immediate increase in amino acid oxidation already after a modest protein intake (Fig. 2) [11].

**Fig. 3:**
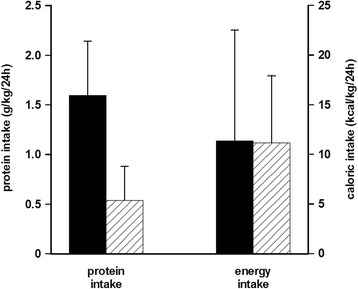
Estimated intake of protein (as calculated from the nitrogen intake × 6.25) and energy from published data in the REDOXS study. *Filled bars* represent patients given glutamine dipeptide supplementation and *hatched bars* represent patients not given extra glutamine dipeptide supplementation. From published data in the REDOXS study [35]

As an example, the results of the REDOXS study (including high-dose glutamine-dipeptide supplementation) may be discussed in terms of protein intake [35, 36]. The main result of the study was harm associated with high-dose glutamine supplementation. The nitrogen intakes correspond to a protein intake of 0.50 g/kg/24 h and 1.56 g/kg/24 h in the two groups, with a concomitant energy intake of 11 kcal/kg/24 h (Fig. 3). In the REDOXS trial the nitrogen intake was not in the form of a high-quality milk protein but a highly unbalanced amino acid mixture of mainly glutamine, alanine, and glycine in dipeptide form. This unbalanced amino acid composition is bound to increase amino acid oxidation. An alternative hypothesis for the results of the REDOXS study may therefore be that a possible elevated rate of amino acid oxidation in parallel to a low energy intake may create a metabolic burden, perhaps metabolic acidosis. So far the post-hoc analysis of the REDOXS trial has suggested an association between compromised kidney function and unfavorable outcomes [36]. This is a group of patients that may be particularly susceptible to a high intake of a low-quality protein, which the body will try to handle by oxidation and ureagenesis.

## Conclusions

Although the concept of enhancing the protein intake in the early phase of critical illness to counteract and attenuate the losses is not far-fetched, the evidence for efficacy of this strategy is not impressive. Historically, efforts to dampen the underlying mechanism of catabolism, such as better pain control, better resuscitation, and more effective infectious control etc., have proven to be effective. More direct interferences with protein metabolism are less convincing. For example, pharmacological doses of growth hormone stimulate muscle protein synthesis, but increase mortality [37, 38]. Post-hoc analyses of data from the EPANIC study make the authors suggest that the statistical correlation between protein intake and outcomes indicate that protein is associated with unfavorable outcomes, possibly by inhibiting autophagy [31, 39].

General recommendations to increase the amount of protein feeding in the acute phase of critical illness may not be the right way to go. The evidence for benefit comes from observational studies, not from prospective randomized studies. The concerns for risks are not sufficiently explored, as the patients at particular risk are often excluded in study protocols. For the majority of patients, a doubling of the recommended protein intake from 1.2–1.5 g/kg/24 h to 2.5–3.0 g/kg/24 h may not be harmful, but the efficacy is still to be demonstrated. For other patients with various forms of limited physiological reserves in terms of vital organ functions, the current upper limit, according to ESPEN guidelines, of 1.5 g/kg/24 h may already create problems. Therefore, we would advocate a more conservative attitude in general recommendations pending creation of more solid knowledge. We see no rationale for a higher protein intake than the ESPEN guidelines recommendation, even if whole body protein balance is more positive as our own studies demonstrate [11, 13–15], unless clinical relevant advantages can be demonstrated in randomized studies. In parallel, we need to better identify the possible risks involved with a high-protein intake on the level of the individual patient. What will be the optimal intake for a patient with a high nutrition risk and simultaneously compromised kidney and liver functions?

## Abbreviations

BMI: Body mass index
CONSORT: Consolidated Standards of Reporting Trials
EPANIC: Early versus Late Parenteral Nutrition in Critically Ill Adults
ICU: Intensive care unit
RCT: Randomized controlled trials
REDOXS: REducing Deaths due to OXidative Stress

## References

[1] Weijs PJ, Looijaard WG, Dekker IM, Stapel SN, Girbes AR, Oudemans-van Straaten HM, Beishuizen A (2014). Low skeletal muscle area is a risk factor for mortality in mechanically ventilated critically ill patients. Crit Care.

[2] Herridge MS, Tansey CM, Matte A, Tomlinson G, Diaz-Granados N, Cooper A, Guest CB, Mazer CD, Mehta S, Stewart TE (2011). Functional disability 5 years after acute respiratory distress syndrome. N Engl J Med.

[3] Prado CM, Gonzalez MC, Heymsfield SB (2015). Body composition phenotypes and obesity paradox. Curr Opin Clin Nutr Metab Care.

[4] Gamrin L, Andersson K, Hultman E, Nilsson E, Essen P, Wernerman J (1997). Longitudinal changes of biochemical parameters in muscle during critical illness. Metabolism.

[5] Puthucheary ZA, Rawal J, McPhail M, Connolly B, Ratnayake G, Chan P, Hopkinson NS, Padhke R, Dew T, Sidhu PS (2013). Acute skeletal muscle wasting in critical illness. JAMA.

[6] Leverve X, Guignier M, Carpentier F, Serre JC, Caravel JP (1984). Effect of parenteral nutrition on muscle amino acid output and 3-methylhistidine excretion in septic patients. Metabolism.

[7] Allingstrup MJ, Esmailzadeh N, Wilkens Knudsen A, Espersen K, Hartvig Jensen T, Wiis J, Perner A, Kondrup J (2012). Provision of protein and energy in relation to measured requirements in intensive care patients. Clin Nutr.

[8] Alberda C, Gramlich L, Jones N, Jeejeebhoy K, Day AG, Dhaliwal R, Heyland DK (2009). The relationship between nutritional intake and clinical outcomes in critically ill patients: results of an international multicenter observational study. Intensive Care Med.

[9] Elke G, Wang M, Weiler N, Day AG, Heyland DK (2014). Close to recommended caloric and protein intake by enteral nutrition is associated with better clinical outcome of critically ill septic patients: secondary analysis of a large international nutrition database. Crit Care.

[10] Essen P, McNurlan MA, Gamrin L, Hunter K, Calder G, Garlick PJ, Wernerman J (1998). Tissue protein synthesis rates in critically ill patients. Crit Care Med.

[11] Rooyackers O, Kouchek-Zadeh R, Tjader I, Norberg A, Klaude M, Wernerman J (2015). Whole body protein turnover in critically ill patients with multiple organ failure. Clin Nutr.

[12] Klaude M, Mori M, Tjader I, Gustafsson T, Wernerman J, Rooyackers O (2012). Protein metabolism and gene expression in skeletal muscle of critically ill patients with sepsis. Clin Sci (Lond).

[13] Berg A, Rooyackers O, Bellander BM, Wernerman J (2013). Whole body protein kinetics during hypocaloric and normocaloric feeding in critically ill patients. Crit Care.

[14] Liebau F, Sundstrom M, van Loon LJ, Wernerman J, Rooyackers O (2015). Short-term amino acid infusion improves protein balance in critically ill patients. Crit Care..

[15] Liebau F, Wernerman J, van Loon LJ, Rooyackers O (2015). Effect of initiating enteral protein feeding on whole-body protein turnover in critically ill patients. Am J Clin Nutr.

[16] Ishibashi N, Plank LD, Sando K, Hill GL (1998). Optimal protein requirements during the first 2 weeks after the onset of critical illness. Crit Care Med.

[17] Dickerson RN, Pitts SL, Maish GO, Schroeppel TJ, Magnotti LJ, Croce MA, Minard G, Brown RO (2012). A reappraisal of nitrogen requirements for patients with critical illness and trauma. J Trauma Acute Care Surg.

[18] van Schijndel RJ S, Weijs PJ, Koopmans RH, Sauerwein HP, Beishuizen A, Girbes AR (2009). Optimal nutrition during the period of mechanical ventilation decreases mortality in critically ill, long-term acute female patients: a prospective observational cohort study. Crit Care.

[19] Weijs PJ, Looijaard WG, Beishuizen A, Girbes AR, Oudemans-van Straaten HM (2014). Early high protein intake is associated with low mortality and energy overfeeding with high mortality in non-septic mechanically ventilated critically ill patients. Crit Care.

[20] Nicolo M, Heyland DK, Chittams J, Sammarco T, Compher C (2016). Clinical outcomes related to protein delivery in a critically ill population: a multicenter, multinational observation study. JPEN J Parenter Enteral Nutr.

[21] Compher C, Chittams J, Sammarco T, Nicolo M, Heyland DK (2017). Greater protein and energy intake may be associated with improved mortality in higher risk critically ill patients: a multicenter, multinational observational study. Crit Care Med.

[22] Larsson J, Lennmarken C, Martensson J, Sandstedt S, Vinnars E (1990). Nitrogen requirements in severely injured patients. Br J Surg.

[23] Ferrie S, Allman-Farinelli M, Daley M, Smith K (2016). Protein requirements in the critically ill: a randomized controlled trial using parenteral nutrition. JPEN J Parenter Enteral Nutr.

[24] Doig GS, Simpson F, Bellomo R, Heighes PT, Sweetman EA, Chesher D, Pollock C, Davies A, Botha J, Harrigan P (2015). Intravenous amino acid therapy for kidney function in critically ill patients: a randomized controlled trial. Intensive Care Med.

[25] Verbruggen SC, Coss-Bu J, Wu M, Schierbeek H, Joosten KF, Dhar A, van Goudoever JB, Castillo L (2011). Current recommended parenteral protein intakes do not support protein synthesis in critically ill septic, insulin-resistant adolescents with tight glucose control. Crit Care Med.

[26] Geukers VG, Dijsselhof ME, Jansen NJ, Breur JM, van Harskamp D, Schierbeek H, van Goudoever JB, Bos AP, Sauerwein HP (2015). The effect of short-term high versus normal protein intake on whole-body protein synthesis and balance in children following cardiac surgery: a randomized double-blind controlled clinical trial. Nutr J..

[27] Casaer MP, Mesotten D, Hermans G, Wouters PJ, Schetz M, Meyfroidt G, Van Cromphaut S, Ingels C, Meersseman P, Muller J (2011). Early versus late parenteral nutrition in critically ill adults. N Engl J Med.

[28] Allingstrup MJ, Kondrup J, Wiis J, Claudius C, Pedersen UG, Hein-Rasmussen R, Jensen TH, Lange T, Perner A. Early goal-directed nutrition in ICU patients (EAT-ICU): protocol for a randomised trial. Dan Med J. 2016;63(9). 27585532

[29] Rugeles S, Villarraga-Angulo LG, Ariza-Gutierrez A, Chaverra-Kornerup S, Lasalvia P, Rosselli D (2016). High-protein hypocaloric vs normocaloric enteral nutrition in critically ill patients: a randomized clinical trial. J Crit Care..

[30] Sundstrom Rehal M, Tjader I, Wernerman J (2016). Nutritional needs for the critically ill in relation to inflammation. Curr Opin Clin Nutr Metab Care.

[31] Casaer MP, Wilmer A, Hermans G, Wouters PJ, Mesotten D, Van den Berghe G (2013). Role of disease and macronutrient dose in the randomized controlled EPaNIC trial: a post hoc analysis. Am J Respir Crit Care Med.

[32] Dvir D, Cohen J, Singer P. Computerized energy balance and complications in critically ill patients: an observational study. Clin Nutr. 2006;25:37–44. 10.1016/j.clnu.2005.10.010 16321459

[33] Villet S, Chiolero RL, Bollmann MD, Revelly JP, Cayeux RNM, Delarue J, Berger MM (2005). Negative impact of hypocaloric feeding and energy balance on clinical outcome in ICU patients. Clin Nutr.

[34] Hoffer LJ, Bistrian BR (2012). Appropriate protein provision in critical illness: a systematic and narrative review. Am J Clin Nutr.

[35] Heyland D, Muscedere J, Wischmeyer PE, Cook D, Jones G, Albert M, Elke G, Berger MM, Day AG (2013). A randomized trial of glutamine and antioxidants in critically ill patients. N Engl J Med.

[36] Heyland DK, Elke G, Cook D, Berger MM, Wischmeyer PE, Albert M, Muscedere J, Jones G, Day AG, on behalf of the Canadian Critical Care Trials G. Glutamine and antioxidants in the critically ill patient: a post hoc analysis of a large-scale randomized trial. J Parenter Enteral Nutr. 2015;39:401–9. 10.1177/0148607114529994 PMC6800175 24803474

[37] Gamrin L, Essen P, Hultman E, McNurlan MA, Garlick PJ, Wernerman J (2000). Protein-sparing effect in skeletal muscle of growth hormone treatment in critically ill patients. Ann Surg.

[38] Takala J, Ruokonen E, Webster NR, Nielsen MS, Zandstra DF, Vundelinckx G, Hinds CJ (1999). Increased mortality associated with growth hormone treatment in critically ill adults. N Engl J Med.

[39] Casaer MP, Van den Berghe G (2014). Nutrition in the acute phase of critical illness. N Engl J Med.

